# King George's Fund for Sailors

**Published:** 1917-11-17

**Authors:** Randall Cantuar

**Affiliations:** Lambeth Palace.


### King George's Fund for Sailors.

We have received from Captain A. W. Clarke, Deputy Chairman of this Fund, a letter conveying the thanks of the General Council to THE HOSPITAL for "the large measure of assistance it has rendered to this Fund." We have further received a copy of a letter from the Archbishop of Canterbury, which we append :—

DEAR CAPTAIN CLARKE,—I entirely share the opinion which has been finding expression to the effect that in the eager endeavour which we have rightly made to further the interests and promote the comfort of our soldiers there has been a tendency to give inadequate recognition and aid to the efforts on behalf of our sailors, which have an equal claim upon us and which it should be our privilege to further in every way that we can. I have therefore rejoiced in the establishment of King George's Fund for Sailors, formed not merely to prevent the overlapping of existing agencies but to stimulate the promotion of fresh endeavour for the aid and comfort of seamen and their dependents. It has been appropriately suggested that to a central Fund, thus inaugurated as King George's Fund for Sailors with His Royal Highness Prince Albert as President, the congregations in all our churches should have an opportunity of contributing. In my judgment this is absolutely right, and I hope you will take such steps as are necessary for inviting the clergy and churchwardens in all our parishes, where it is possible to do so, to make arrangements accordingly. The public ought to be aroused to a full recognition of what the country and the Empire owe to the courage and contempt of danger shown by the seamen of the Royal Navy, and not less notably by the equally gallant men of the Merchant Service in mail and cargo vessels, in minesweepers, mine-layers, and patrol boats, both on our coasts and far out at sea.

If for necessary reasons it is impossible at present to make public the details of what is daily being done, the time will come when our eyes will be opened to the magnitude of the effort and the depth of the devotion of these men, and we shall be glad to feel that we have from the first undertaken what is necessary on behalf of them and their families. With all my heart do I wish God-speed to your appeal, and I shall lose no opportunity of urging its importance and its significance.—I am, yours very truly, (Signed) RANDALL CANTUAR.

Lambeth Palace.

[Last week's meeting is reported on page 151. A note upon the proposed Sailors' Sunday for the benefit of the Fund occurs in our News columns.—ED. THE HOSPITAL.]

